# Effect of Agriculture and Construction Wastes on the Properties of Magnesium Oxychloride Cement Mortar with Tourmaline Powder

**DOI:** 10.3390/ma12010115

**Published:** 2018-12-31

**Authors:** Xue-Fei Chen, Shi-Cong Kou, Feng Xing

**Affiliations:** 1 School of Materials Science and Engineering, Nanjing University of Science and Technology, Nanjing 210094, China; chenxuefei@email.szu.edu.cn; 2 Department of Civil Engineering, Shenzhen University, Shenzhen 518000, China

**Keywords:** magnesium oxychloride mortar, clay brick power, corn stalk, saw dust, tourmaline power

## Abstract

This research attempted to develop an environmentally-friendly functional building mortar by the combined use of agriculture wastes (agro-wastes) and construction wastes in magnesium oxychloride cement (MOC). The agro-wastes referred to corn stalk (CS) and saw dust (SD), which were used to improve the flexural properties of host cementitious material, whilst the construction wastes referred to recycled clay brick powder (CBP), which was employed to enhance compressive strength and water resistance. Moreover, tourmaline powder (TP) was added as a negative ion-inducing admixture, at a fixed dosage of 10% by weight of MgO, to bestow air-improving functions on the end products. Results showed the flexural strength of MOC was enhanced by the addition of CS and SD. Besides, the incorporation of CBP improved the water resistance in a dosage-dependent way. In addition, the specimens containing CS and SD also had better negative ion-inducing performance due to their higher porosity. Overall, the study provided a feasible and attractive approach to recycle agro- and construction wastes for the production of air purifying mortar. The developed mortar possesses an eco-friendly nature (simultaneous reuse of various waste materials and improvement of the air quality).

## 1. Introduction

Magnesium oxychloride cement (MOC) is an air-dried magnesia-based cementing material that was developed shortly after ordinary Portland cement (OPC). It is superior to OPC with respect to its rapid setting and hardening properties, excellent abrasion resistance, and good bonding capacity to hold large amounts of various inert fillers [1,2]. Besides, MOC consumes less energy, and releases less carbon dioxides during its production, since a reduced calcination temperature is required. Thus, MOC is more environmentally friendly and sustainable. However, a crucial defect of MOC is its weak water resistance. That means the mechanical strength drops when MOC suffers from a water attack [3]. It has been reported that MOC suffers a significant loss in its compressive strength when it is immersed in water for 28 days. The decomposition of Phase 5 (5Mg(OH)_2_·MgCl_2_·8H_2_O) and Phase 3 (3Mg(OH)_2_·MgCl_2_·8H_2_O), which are the main contributors of strength, was responsible for the observed strength decline [4]. Thus, extensive studies have been conducted, to address the problem of MOC’s water solubility. In particular, additives represent an effective way to improve MOC’s water resistance. For example, the addition of phosphate in MOC effectively prevents the magnesium cement crystal from decomposing, due to the formation of insoluble hydrated products (such as magnesium phosphate) [5]. Meanwhile, it has also been pointed out that the phosphate-induced improvement in the water resistance of MOC can be attributed to the transformation from the crystalline to gel-like Phase 5 [6]. Moreover, Li et al. found that the mechanical properties of MOC are also improved by adding granite wastes [5], pulverized fuel ash [7], incinerated sewage sludge [8], glass powder [9], and fly ash [4] into MOC, since these materials generate a filling effect and gels under alkaline conditions. Interestingly, fly ash is found to be a promising waste material, which not only significantly improves the water resistance, but also the residual compressive strength of MOC [4]. Despite the effectiveness of additives in improving the properties (especially the water resistance) of MOC, the cost of the end-materials is unfavorably increased. Thus, alternative waste materials such as recycled clay brick powder (CBP) hold great promise for improving the properties of MOC in a cost-effective way, because CBP is crushed from recycled clay bricks, and is a typical pozzolanic material, and has similar properties to grade II fly ash [10]. Specifically, CBP is recorded to mainly constitute free silica quartz and amorphous aluminosilicate, as well as albite and hematite [11] which are beneficial for enhancing the water resistance of MOC. It enlightens us to incorporate CBP into MOC, to improve the water resistance of MOC. Actually, recycled clay bricks (RCBs) represents a considerable proportion [12] in the construction and demolition wastes (C&DW), of which billions of tons are generated each year, due to the rapid urbanization and renovation of old cities [13]. However, the extreme high porosity [14] and the associated high crushing index greatly limits their use in the form of aggregates. Typically, RCBs are landfilled, exerting a heavy burden on precious land resources. Thus, to blend CBP into MOC holds great promise for improving the properties of MOC in a cost-effective way. 

Similar to OPC, MOC is also a typical brittle material. It means that MOC can only suffer much lower flexural and tensile strength in comparison to its compressive strength. An effective approach to improving the defect is to incorporate fibers, to increase the toughness. Common reinforcement fibers are glass fibers, carbon fibers, and steel fibers. However, those fibers are all more expensive and heavy than natural fibers retrieved from agro-waste. Actually, besides the C&DWs, agro-waste is another typical composition of solid wastes. Corn stalk (CS) [15] and saw dust (SD) are two common agro-wastes in China. To be specific, the corn stalk refers to the part of the corn plant remaining after the seed is harvested. Traditionally, corn stalks are directly burned in cropland, causing serious air pollution. The sawdust refers to the dust-like materials that are generated during the cutting process of woods and wood products. In most cases, sawdust is left where it is produced. However, it might trigger respiratory problems, since it is small and easy to inhale. To deal the above problems, previous researchers have incorporated agro-wastes into cements, and have found they can enhance the insulation for building materials [16,17], function as reinforcement fibers [18,19,20], and have good adaptability with cement [21]. Thus, the research immobilizes these agro-wastes with cementitious materials, expecting to reduce the cost and density of building materials, and at the same time improve the flexural strength by relying on the fiber-reinforcement effect. Particularly, the cementitious material of MOC compared with OPC holds a higher mechanical strength. It endows MOC with a higher upper threshold, to accommodate inert fillers, namely CS, SD, and the recycled clay brick powders addressed above. 

At length, tourmaline powder is added into MOC, to endow the final products with an air-purifying function, and to increase the usage value of the prepared building material. Tourmaline powder is the mechanical grinding powder of raw tourmaline ore after the removal of impurities. Once processed and purified, it has a high negative ion yield and far infrared emissivity. The general chemical formula of tourmaline is XY_3_Z_6_(T_6_O_18_)(BO_3_)V_3_W_3_. The crystal belongs to a group of ring-structured silicate minerals of the rhombohedral system. R stands for a metal cation. When R is Fe^2+^, the black crystal tourmaline is formed. Tourmaline crystal is nearly triangular columnar shaped, and has different crystals at both ends. In particular, the tourmaline powder is piezoelectric and thermoelectric. Overall, the tourmaline powder is a natural mineral that relies on its self-polarization effect to induce negative ions in the air [22], whilst the concentration of negative ions is a crucial indicator of fresh air, because it purifies air and promotes physical and mental health [23]. 

To this end, this research develops an environmentally-friendly building mortar from agro-wastes of CS and SD, construction wastes of CBP, tourmaline powder, and MOC. Physical properties, including water absorption, specific density, compressive strength, and flexural strength were determined first. Afterwards, the water resistance was tested. At last, the negative ion-inducing capacity was measured. The developed mortar was expected to be used for both internal and external applications. 

## 2. Experimental Details

### 2.1. Materials

**Figure 1:**
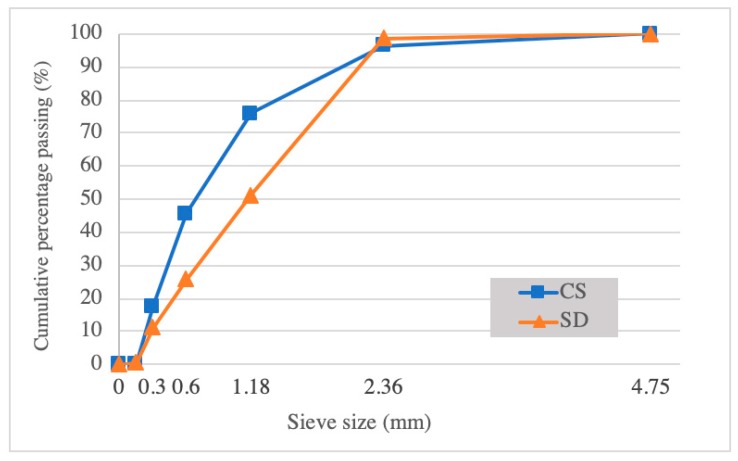
Sieving curves of CS and SD.

MgO (95% purity, density of 1550 kg/m^3^) and MgCl_2_·6H_2_O (96% purity) are purchased from Kuner Chemical Industry (Guangzhou, China). The CBP (less than 45 μm) was obtained by milling the recycled clay bricks supplied by a local building materials company in Shenzhen. The CS and SD were provided by Shengxiang Environmental Technology Company (Jilin, China). The tourmaline powder (TP) (less than 45 μm) was purchased from market. All of the water used in this study was tap water. Table 1 (provided by Shengxiang Environmental Technology Company, Jilin, China), Table 2, and Figure 1, respectively illustrate the basic chemical and physical properties and the sieving curve of CS and SD. The chemical composition of CBP and TP was determined by using an X-ray fluorescence spectrometer (Model: S4 Explorer, Bruker AXS GmbH, Heidelberg, Germany). The density and water absorption were determined as per JGJ 52-2006, Chinese standard. Hereafter, the CSA and SDA respectively indicate the corn stalk aggregate and the saw dust aggregate. 

### 2.2. Sample Preparation

Table 3 shows the mix proportions of the mortar samples prepared. A total of 10 mixtures were prepared. All specimens were cured in an environmental chamber at 25 °C, and a relative humidity (RH) of 55%. CBP was added as an additive at dosages of 15%, 25%, and 35% by mass of MgO. CS and SD were added at dosages of 5%, 10%, and 15% by mass of MgO, to improve the flexural strength. TP was added as a negative ion-releasing admixture at a fixed dosage of 10% by mass of MgO. 

**Figure 2:**
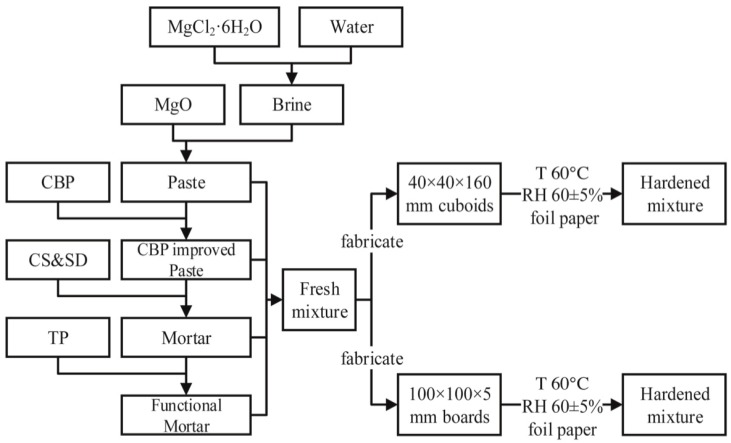
Sample preparation procedures.

Figure 2 illustrates the main procedures of the sample preparation. The required amount of magnesium chloride was first dissolved in tap water and thoroughly mixed for about 1 min in a mechanical mixer. Afterwards, MgO powder, CBP, and CS/SD were added and further mixed for about 3 min. The prepared materials were then cast into steel molds. The specific gravity, water resistance, and mechanical properties of MOC were determined on prismoid specimens (40 × 40 × 160 mm). Meanwhile, the water absorption and negative ion-inducing properties of MOC were measured on board specimens (100 × 100 × 5 mm). After one day of casting, the specimens were demolded and cured in air for 3, 7, and 28 days, respectively. 

### 2.3. Test Methods

#### 2.3.1. Fluidity

**Figure 3:**
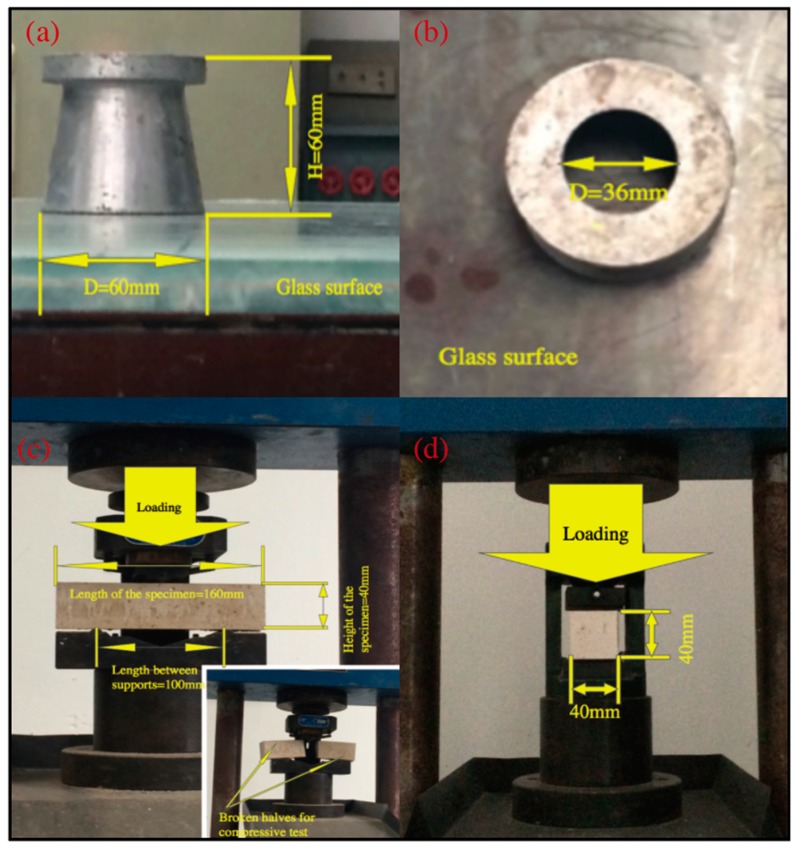
(**a**) Horizontal view of the cone-type mold for the fluidity test; (**b**) vertical view of the cone-type mold for the fluidity test; (**c**) three-point bending test; (**d**) the uniaxial compressive test.

The fluidity of the samples was tested using the flow table method according to GB/T2419-2005 [24]. The average flow diameter of a conical frustum formed by a cone-type mold was measured after 30 s on a glass surface (Figure 3a). Three samples were tested for each composition, and the average results were reported.

#### 2.3.2. Specific Gravity

The specific gravity of the specimens was tested using a water displacement method, as per Archimedes’ principle. First, the container was filled with water. Then, the specimen with a 28-day curing age was weighed (denoted as DW). After that, the weighted specimen was immersed in water with a string. The weight of the specimen in water was measured (denoted as WW). Each of the reported values was the average of three samples. The specific gravity was then calculated by Equation (1):

(1) $$\begin{array}{c} \frac{DW}{\left(DW-WW\right)}\times G_{w25℃} \end{array}$$

where the $G_{w25℃}$ is the specific gravity of pure water at 25 °C. The exact value is 0.99705 g/cm^3^, which was appropriately set as 1 g/cm^3^. 

#### 2.3.3. Water Absorption

The 48 h water absorption tested was conducted as follows. First, the prepared specimens were oven-dried at 105 ± 5 °C to a constant weight. Second, the oven-dried specimens were placed into a glass drier to gradually cool to room temperature. The dry weight was denoted as G_0_. Third, the dry specimens were placed into a water tank, and separated from the bottom of water tank by glass rods. After 48 h, the immersed specimen was taken out and wiped by a wet towel to remove the water on the surface. The wet weight was denoted as G_s_. The water absorption (Wa) was calculated by Equation (2):

(2) $$\begin{array}{c} W_{a}=\frac{G_{s}-G_{0}}{G_{0}} \end{array}$$

The final water absorption was recorded as the maximum value of three tests. 

#### 2.3.4. Flexural strength

The three-point flexural strength test in conformity with GB/T17671-1999 [25] was conducted after seven days of curing. The prism specimens were tested in a universal testing machine (50 KN) under a central line load, while simply being supported over a span of 120 mm (Figure 3c). The specimens were then loaded at a displacement rate of 0.20 mm/min until failure. The results reported are the average of three specimens.

The flexural strength (R_f_) was calculated by Equation (3):

(3) $$R_{f}=\frac{3PL}{2bh^{2}}$$

where, R_f_ (MPa) indicates the flexural strength of the specimen; P (N) indicates to the loading when the specimen breaks; L (mm) indicates to the center distance between two bottom supporters; b indicates to width (mm) of the specimen; h (mm) indicates to the height of the specimen. 

#### 2.3.5. Compressive Strength

As per GB/T17671-1999 [25], the compressive strength was determined by using conventional compression with a load capacity of 3000 kN on the broken pieces (portions of the prisms broken in the flexure strength test) (Figure 3d) at 3, 7, and 28 days. The reported test results are the average of six measurements.

The compressive strength (Rc) was calculated by Equation (4):

(4) $$R_{c}=\frac{P}{A}$$

where Rc (MPa) is the compressive strength of the specimen; P (N) is the loading when the specimen breaks; A (mm^2^) is the effective loading area of the specimen, namely 1600 mm^2^. 

#### 2.3.6. Water Resistance

The water resistance test was available for specimens with CBP. In this paper, the water resistance performance was reflected by the water resistance index (WRI), which was calculated by Equation (5). In particular, the WRI was developed by the authors, since no standard reference was available to determine the water resistance:

(5) $$\frac{compressive\;strength\;of\;specimen\;A}{compressive\;strength\;of\;specimen\;B}\times 100\%$$

where specimen A was prepared by curing in a standard curing chamber (temperature 60 °C, relative humidity 60 ± 5%) for 14 days, followed by curing in water for another 14 days under the same curing conditions; specimen B was prepared by curing in the same standard curing chamber for 28 days. Each of the reported values is the average of three samples. 

#### 2.3.7. Negative Induction

The specimen was first dried in an oven under (60 ± 2) °C for 1 h. Then, the specimen was allowed to cool down gradually at ambient temperature. After 24 h, the inducing negative air ion concentration was calculated, according to Equation (6):

(6) C=A−B

where *C* stands for the effective inducing negative air ion concentration of specimens; *A* stands for the tested induced negative air ion concentration after the placement of specimens; *B* stands for the tested induced negative air ion concentration before the placing of specimens, i.e., the induced negative air ion concentration of the base air. The units of *A*, *B*, and *C* are ion/s·cm^2^, which stands for the amount of ions generated in the air over 1 s per square centimeter.

**Figure 4:**
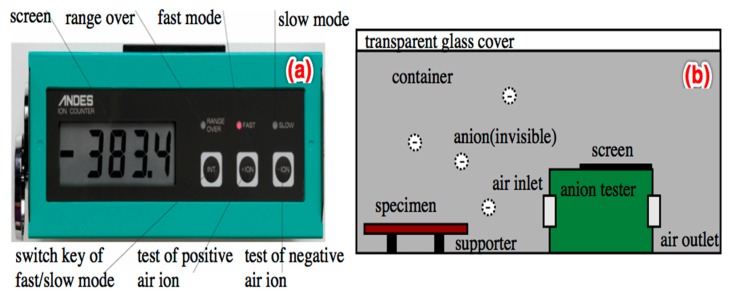
NT-C101A and the self-made test equipment of anions.

Specifically, *A* and *B* were tested according to the method of dynamic testing on air ions, as per JC/T1016-2006 Testing, on the negative ion concentration of materials. The method forced the air to go through an anion collector, and tested the concentration of negative air ions yielded. In this study, the method was performed using a commercial anion tester, denoted as NT-C101A (Figure 4a), and self-made testing equipment (Figure 4b). In particular, the NT-C101A was a tester manufactured according to Japanese Industrial Standards (JIS) and which satisfied requirements as per GB/T 18809-2002 General specifications for air ion measuring instruments, and which was capable of testing both the concentrations of the positive and negative air ions. 

To ensure that no air leaks outside the container, the equipment was closely sealed by rubbers on edges, where the transparent glass cover was connected with the main container. Before testing, the testing machine was turned on for 5 min to ensure that it reached its best working status. Then, the board specimens cured in the standard chamber for 28 days were placed on the supports located at the bottom of the container. Next, the glass cover was closed, and the amount of negative ions in the container was directly read by every 6 min from the screen of the test machine. Inducing negative air ion concentrations designated as A in Equation (6) was calculated by Equation (7):

(7) $$A\left(ion\cdot s^{-1}\cdot cm^{-2}\right)=\;\frac{S\times V}{T\times Area}$$

where *S* stands for the accumulated amount of negative ions for all times of reading (unit ion/cc.); *V* stands for the internal volume (unit cm^3^) of the container; *T* stands for the times of reading (*T* is 11 in this report because the testing period is 1 h); Area stands for the full surface area (unit cm^2^) of the specimen. 

As to the variable of *B* in Equation (6), it was tested by following the same procedures shown above. However, the variable of *Area* in Equation (7) was changed to the base area of the container shown in Figure 4b. The final result was the average of three tests. 

In China, a specific national standard associated with anion-improved construction materials is JC/T 2040-2010: Indoor decorative materials with the function of negative air ions. According to the standard, the negative air ion- induced concentration of indoor decorative materials with the function of inducing negative air ions should be no less than 500 ion/s·cm^2^ under a room temperature of 20~25 °C and a relative humanity of 20~40%. Furthermore, the air-negative oxygen ion concentration is graded into six gradations (Table 4), as per the LY/T 2586-2016 Specification on the observation of air negative oxygen ion concentration. 

## 3. Results and Discussion

### 3.1. Fluidity

**Figure 5:**
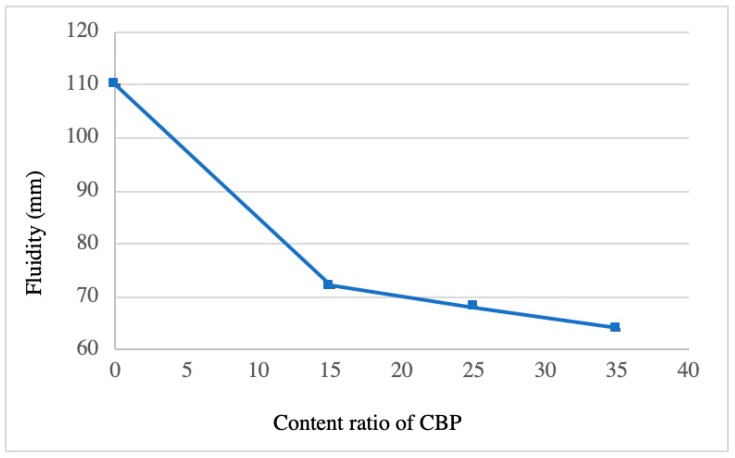
Influence of CBP on the fluidity of fresh mixtures. The content ratio of CBP means the weight percentage of MgO.

Figure 5 shows the influence of CBP on the fluidity of the freshly blended mixtures. The fluidity of the specimens decreased as the content of CBP increased. This was mainly caused by two CBP-induced effects. One was the higher water absorption ability of CBP (see Table 1), which resulted in a reduction of the free water. The other was the irregular CBP surface (a high roughness) [11], which was adverse to the movement between the mixture particles. The result was also verified by other research that concluded that the irregular microstructure of recycled power demands more water-reducing agent to maintain the required workability of the mortar. Notably, the influence of CBP on the fluidity was more significant when its content was less than 15%. In other words, the fluidity dropped significantly with increasing CBP from 0 to 15%. In contrast, a further increase of CBP from 15% to 35% led to a negligible decrease in fluidity. This may be because at the water-to-binder ratio used, most of the free water in the mixture was already absorbed by CBP at a 15% replacement ratio. A further alleviated decline in fluidity induced by CBP (above 15%) was likely due to its pore-filling effect, which inhibited the movement of the mixed particles. Almost no fluidity existed when the content of CBP was increased to 35%. As for the mortars, namely pastes containing SDA and CSA, the flow table method was used to determine the fluidity. However, after 25 times vibration, the specimens containing SD and CS had a low fluidity that was hardly recorded. 

### 3.2. Specific Gravity

**Figure 6:**
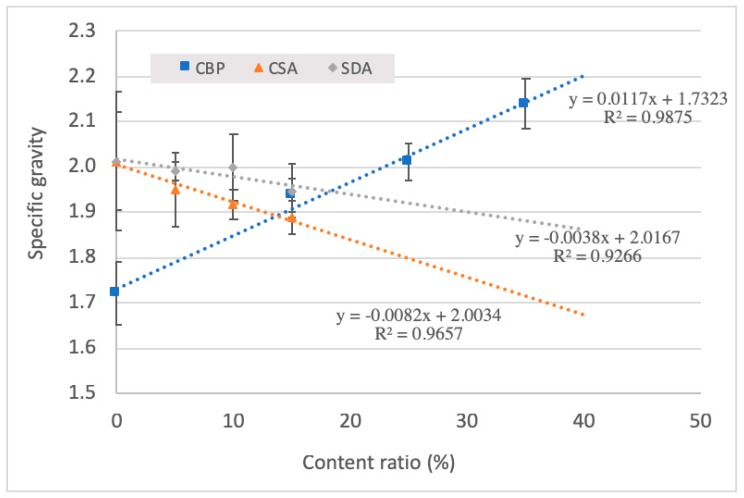
Specific gravity of hardened mixtures incorporated with different contents of CBP, CSA, and SDA. 1. the error bar represents the standard deviation; 2 the content ratio means the weight percentage of MgO.

Figure 6 shows the influence of CBP, CS, and SD on the specific gravity of the hardened mixtures. Evidently, the specific gravity increased as the content of CBP enhanced, due to the higher density of CBP. For the samples containing 25% CBP, increasing the content of CS and SD led to a decrease in the specific gravity. Considering the fixed volume of each mold, the quantity of magnesium oxychloride cement was inevitably reduced once the SD or CS were blended into the mortar. Since the DS and CS have relative lower densities, the specific gravity of the final product declined. Compared with SD, CS induced a more pronounced reduction in the specific gravity. This was attributed to the smaller density of CS relative to SD (see Table 1). For example, adding 15 wt % CS to the cement mortars resulted in a 20% reduction in the specific gravity. Thus, the incorporation of CS in the mortar samples was more advantageous to produce lightweight building materials. The results were consistent with previous research, which highlights that the incorporation of agro- or bio-wastes is beneficial for preparing lightweight building materials, due to their low density [15,26,27,28]. In particular, it can be seen that for all the scatter figures, linear trendlines were fitted. The results show that all the trendlines were well-fitted to those scatter points, since the R^2^ values of all fitted lines were higher than 0.9. Therefore, the increase or decrease of the specific gravity of the specimen seemed to be only associated with the density of the incorporated material. 

### 3.3. Water Absorption and Flexural Strength

**Figure 7:**
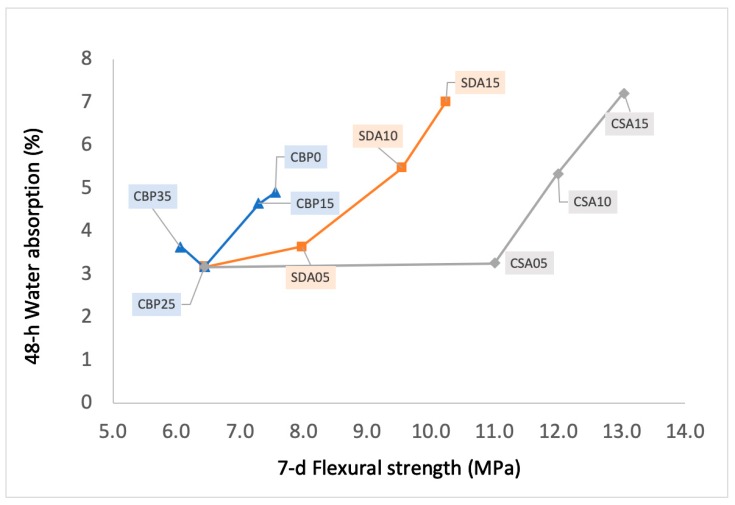
Flexural strength and 48 h water absorption of hardened mixtures incorporated with different contents of CBP, CSA, and SDA.

Figure 7 shows the flexural strength and 48 h water absorption of the hardened mixtures. The flexural strength of the cement mortars decreased with an increase of the content of CBP. The CBP’s impact on the flexural strength was significant. For example, an increase of CBP from 0% to 35% was accompanied by a corresponding 2 MPa (more than 25%) reduction of flexural strength. Apparently, the specimens became denser and more brittle with the incorporation of CBP. An opposite trend was observed for the water absorption. The incorporation of CBP in cement mortars was beneficial for the reduction of water absorption. This is because CBP fills open pores of the specimens, and jointly generates a pozzolanic reaction, thereby blocking water penetration into the mortar matrix. The filler effect is also verified by research that observes a higher microstructure refinement of mortar by the addition of CBP [29]. However, it should be noted that increasing the content of CBP from 25% to 35% leads to a slight increase in the water absorption. Apart from the pore-filling effect, CBP also possesses an inherently high water absorption ability. These two competing effects work together to determine the water absorption of the CBP-incorporated samples. When the content of CBP is below 25%, the pore filling effect plays a dominant role. In contrast, when the content of CBP is further increased beyond 25%, its higher water absorption effect gains the upper hand. 

**Figure 8:**
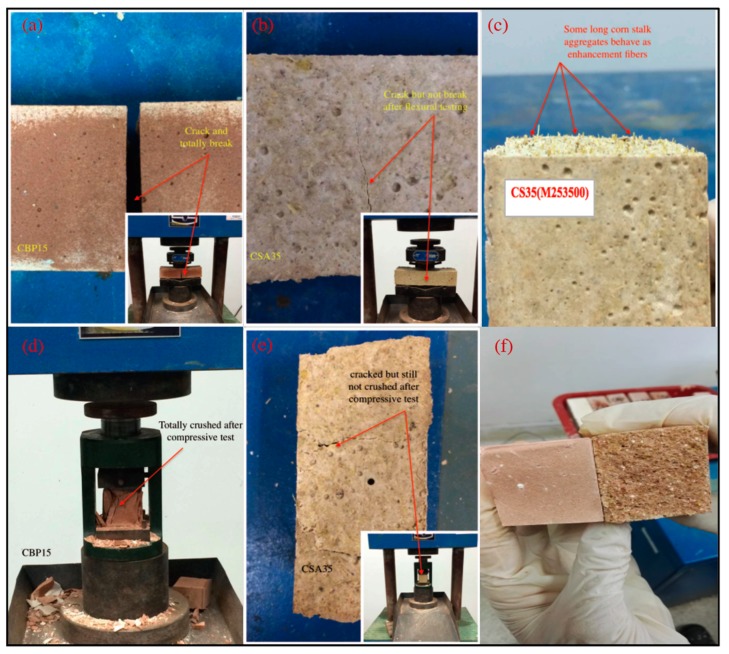
(**a**) flexural test of MCBP15; (b) flexural test of MCSA35; (**c**) horizontal view of MCSA35; (**d**) compressive test of MCBP15; (**e**) compressive test of MCSA35; (**c**) vertical view of MCBP15 (left) and MCSA35 (right).

To CS and SD, both the flexural strength and water absorption of mortars increase when they are blended into mixtures. Furthermore, an increasing content of CS or SD leads to an increase in the flexural strength and water absorption. That CS and SD-induced an increase in the flexural strength was mainly because the blended CS and SD functioned as fibers in the mixture. This explanation is supported by the observations from Figure 8c. It can be seen from Figure 8c that some long CS acts as enhancement fibers in the prepared prismoid. Actually, once CS and SD are added into the matrix, they are in a three-dimensional state that is arbitrarily distributed. Besides, the hardening process of the mortar changes the internal structure of the material, and reduces the internal defect, which improves the continuity of the composite material. This leads the fiber and the matrix to stress together during the loading process [30,31]. In addition, the adhesion effect of the fiber alleviates the load stress concentration of the material, making the matrix crack, but not break, and thus continuously bear extra loads (see Figure 8e). It leads to the increase of tensile strength, and thus, at the same time, enhances the flexural strength. As the consequence, the flexural strength of the material can be significantly improved when the CS and SD are incorporated in appropriate contents. 

The enhanced water absorption of CS- or SD-incorporated mortars is mainly attributed to the expansion of the mixture due to the lower density of CS or SD. In fact, in the magnesium oxychloride system, CS and SD are inert materials and will not participate in the hydration process. Moreover, they are not able to fill the micropores of the mixture, because of their large particle size. Thus, instead of making the cementitious mixtures compact, the incorporation of CS and SD resulted in a slight expansion of the host materials. In addition, the newly formed interfaces between CS (or SD) and the binder matrix became the weak points when subjected to compressive forces. This will be discussed later in this paper. 

### 3.4. Compressive Strength

**Figure 9:**
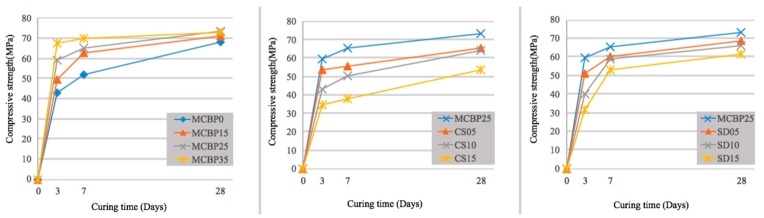
Compressive strength of hardened mixtures incorporated with different contents of CBP, CSA, and SDA.

Compressive strength is always reckoned as a crucial indicator of the quality of cementitious materials. Thus, the compressive strengths of different mixtures are provided in Figure 9. 

As expected, the compressive strengths of all the samples increased with increasing the curing age. Interestingly, all of the mixtures gained the majority of their compressive strengths within the first seven days (especially within the first three days). The results were consistent with the fact that MOC has a high early strength [7,32]. The incorporation of CBP into the MOC enhances the compressive strength in a dosage-dependent way. This can be explained by the following reasons: first, the porous CBP absorbs some free water from the MOC mixture, reducing the effective w/c ratio; second, the small particles of CBP fill the micrometer pores and cracks in the mixture; third, the CBP displays an effect that is similar to a pozzolanic reaction under an alkali condition created by the formation of magnesium hydroxide in the matrix. The effect promotes the generation of a hydrated magnesium silicate gel and magnesium aluminate gels [7], leading to an increase of compressive strength. The first two factors combine to make the mixture denser, while the last factor generates additional gels, and further fills the micropores due to the pozzolanic reaction [4,7]. However, the incorporation of CBP inevitably decreases the effective content of magnesium oxychloride cement in the system. It reduces the crystalline phases that contribute the most to the strength. Nevertheless, in this case (a blending ratio less than 35%), the filling effect and the extra hydrated gel products dominate the strength improvement. This is the hydration product that is produced by active silica and active alumina in CBP is sufficient to offset the decline of strength that is caused by the reduction of the magnesium oxychloride cement.

Unlike CBP, the incorporation of CS and SD in the MOC reduces the compressive strength. This is because CS and SD are inert materials in this magnesium oxychloride system. Thus, when they are blended into the MOC mixtures, the specimens become less compact, due to the weak interfaces between the CS (or SD) and the binder matrix. Also, the addition of CS and SD decreases the cementitious materials, and further reduces the hydration products, leading to the decline of compressive strength.

### 3.5. Water Resistance

**Figure 10:**
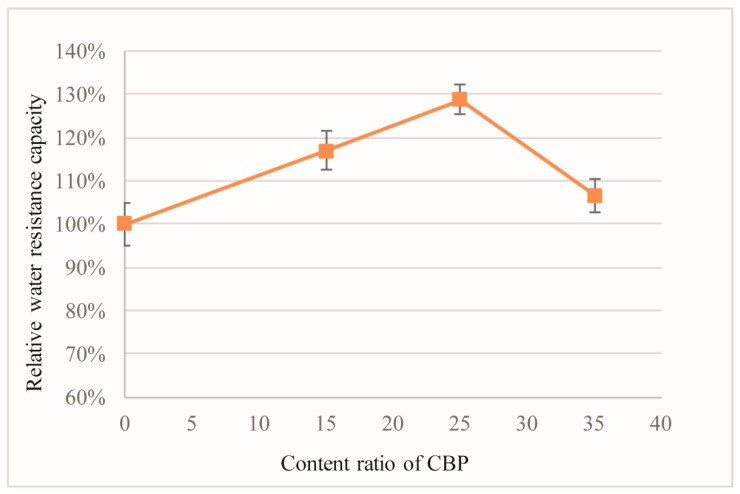
Influence of CBP on the water resistance of hardened mixtures. 1. the error bar represents the standard deviation; 2. the content ratio of CBP means the weight percentage of MgO.

Figure 10 shows the influence of CBP on the water resistance of the MOC. 

All the data are presented as the relative percentage of the water resistance to the control sample. As the content of CBP increases, the water resistance of MOC first increases, and then decreases asymmetrically. The increase in the water resistance is attributed to a denser structure that is induced by the water-blocking effect of CBP, as mentioned above. The blocking effect is also proven by [4,9]. Besides, the improvement of water resistance with CBP incorporation is also due to the amorphous alumino-silicate gel formed by the pozzolanic reaction of the reactive SiO_2_ and Al_2_O_3_ contents of CBP [11] under the alkaline conditions of the MOC system [4,9]. Due to the large surface area, the well-dispersed CBP forms a network of alumino-silicate gel that interacts with the microstructure of the MOC crystals, leading the water-shy MOC phases to be covered to some degree by the CBP surrounded by the insoluble alumino-silicate gels. In contrast, the decrease in the water resistance is caused by the porous structure of CBP (a high porosity) and the associated high water absorption. Overall, when a certain amount of CBP is blended into the MOC mixture, its water resistance performance is improved, beyond that which a decline in the water resistance ensues. In this research, the optimal incorporation content of CBP in the MOC is found to be 25%. 

### 3.6. Negative Ion Induction

**Figure 11:**
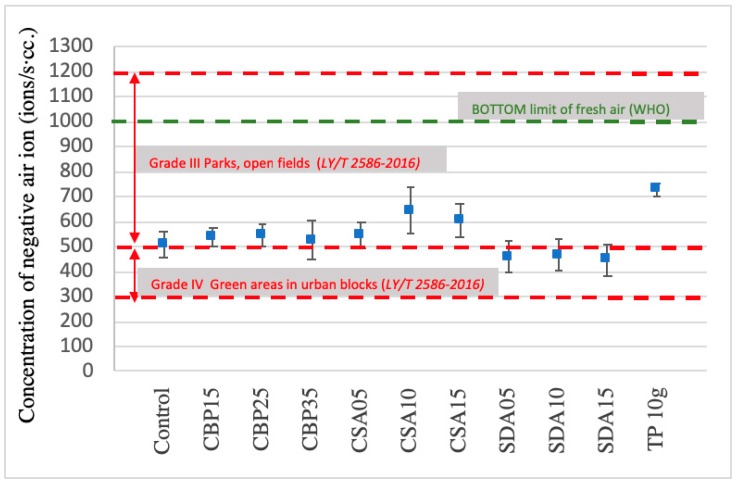
Concentration of negative ions. The error bar represents the standard deviation.

Figure 11 shows the concentrations of negative ions induced by different specimens. Mixtures blended only with CBP are located in Grade IV, whilst the other mixtures are all located in Grade III. 

**Figure 12:**
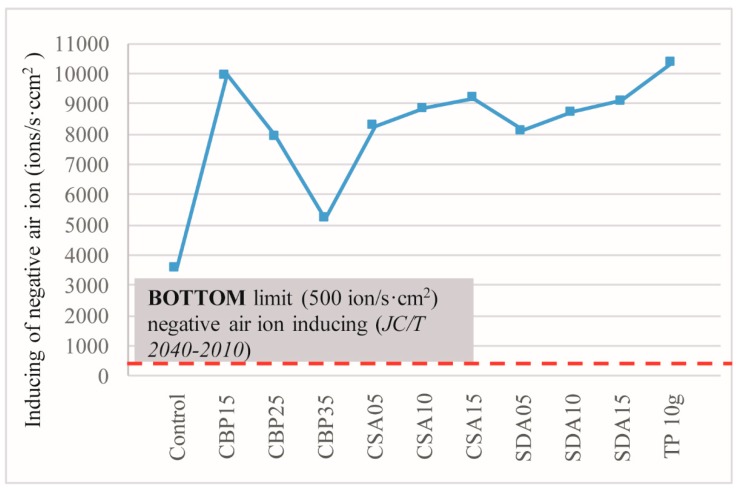
Induction of negative ions.

The concentrations of negative ions induced by all the mixtures are, however, below the bottom limit of fresh air identified by the WHO. Among them, CS is the most beneficial admixture in terms of inducing negative ions, followed by SD and CBP. This is because the CS-blended mixtures have the most incompact structure (reflected by the results of the specific gravity and water absorption). In an incompact structure, more open pores and voids are available, and thus, more air (induced by the TP blended in the mixture) will be accommodated. The result is consistent with previous research that observes that the porous structure of wood promotes negative ion induction [33]. The observation can be explained by two reasons. One is that the high porosity provides a higher contact chance between tourmaline powder, and air and water molecules. It is beneficial because tourmaline relies its weak electric field generated by the spontaneous polarization effect to activate neutral molecules into negative ions [22]. The other reason is that a higher porosity is associated with a larger surface area for tourmaline to load, and thus provides a larger contacting area between tourmaline and air. Figure 12 illustrates the negative ion-inducing capacity of different mixtures. The results mirror the trend of the concentration of negative ions induced by the different specimens identified above. It is noteworthy that all of the specimens fully satisfy the basic requirements for indoor decorative materials, with the function of negative air ions, as per JC/T 2040-2010.

## 4. Conclusions

Based on the findings from this study, the following conclusions can be drawn:

(1) The incorporation of CS and SD in MOC is beneficial to the production of lightweight MOC board, and is also conducive to negative ion induction, because of the incompact structures, in which more voids are available to accommodate more air released by TP.
(2) The incorporation of CBP in MOC improves the water resistance, but reduces the negative ion-inducing ability, due to the denser structure induced by the pore-filling effect and the pozzolanic reaction.
(3) The TP-containing MOC board is able to induce negative ions, thereby improving the air quality of the surrounding environments to a level that is similar to the open fields.

The overall findings demonstrate that the developed MOC mortar holds great potential for practical applications in a sustainable way.

## Figures and Tables

**Table 1:** Basic chemical and physical properties of corn stalk (CS) and saw dust (SD).

Materials	Cellulose	Hemicellulose	Lignin	Extractives	Ash	Tensile Strength	Young Modulus	Elongation at Break	Water Absorption	Bulk Density
	(%)	(%)	(%)	(%)	(%)	MPa	GPa	(%)	(%)	(kg/m^3^)
CS	45.85	21.3	24.2	4	2.7	345	27.6	2.7	12	152.7
SD	37.57	28.56	23.4	8.2	1.2	248	3.2	25	8	195.2

**Table 2:** Basic chemical and physical properties of clay brick powder (CBP) and tourmaline powder (TP).

Materials	SiO_2_	Fe_2_O_3_	Al_2_O_3_	CaO	MgO	SO_3_	Loss on Ignition (LoI)	Water Absorption	Bulk Density
	(%)	(%)	(%)	(%)	(%)	(%)	(%)	(%)	(kg/m^3^)
CBP	53.3	7.4	16.6	6.7	2.5	1.1	6.2	19	1801
TP	40.5	20.42	34.12	0.39	0.59	-	3.98	~0	2500

**Table 3:** Mixture proportions.

Mix. Notation	MgO	MgCl_2_·6H_2_O ^a^	Water ^a^	CBP ^a^	CS ^a^	SD ^a^	TP ^a^
M000000 (M0)	1	0.4	0.3	-	-	-	0.10
M150000 (CBP15)	1	0.4	0.3	0.15	-	-	0.10
M250000 (CBP25)	1	0.4	0.3	0.25	-	-	0.10
M350000 (CBP35)	1	0.4	0.3	0.35	-	-	0.10
M250500 (CS5)	1	0.4	0.3	0.25	0.05	-	0.10
M251000 (CS10)	1	0.4	0.3	0.25	0.10	-	0.10
M251500 (CS15)	1	0.4	0.3	0.25	0.15	-	0.10
M250005 (SD5)	1	0.4	0.3	0.25	-	0.05	0.10
M250010 (SD10)	1	0.4	0.3	0.25	-	0.10	0.10
M250015 (SD15)	1	0.4	0.3	0.25	-	0.15	0.10

^a^. by weight of MgO.

**Table 4:** Gradations of air negative oxygen ion concentration.

Gradation	Concentration of Air Negative Ions	Reference Places	Air Freshness
I	n ≥ 3000	forests, wetland	Best
II	1200 ≤ n < 3000	summits of mountains	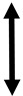
III	500 ≤ n < 1200	parks, open fields	
IV	300 ≤ n < 500	green areas in urban blocks	
V	100 ≤ n < 300	common indoor areas	
VI	n < 100	industrial plants	Worst
